# Urothelial cells may indicate underlying bacteriuria in pregnancy at term: a comparative study

**DOI:** 10.1186/s12884-017-1606-z

**Published:** 2017-12-08

**Authors:** N. Liou, J. Currie, C. James, J. Malone-Lee, A. L. David

**Affiliations:** 1 Institute for Women’s Health, University College London, London, UK; 2 Chronic Urinary Tract Infection Group, Division of Medicine, University College London, London, UK; 3 NIHR University College London Hospitals Biomedical Research Centre, London, UK

**Keywords:** Pregnancy, Urothelium, Urinary tract infection, Sampling method

## Abstract

**Background:**

Urinary tract infection is common in pregnancy. Urine is sampled from by mid-stream collection (MSU). If epithelial cells are detected, contamination by vulvo-vagial skin and skin bacteria is assumed. Outside pregnancy, catheter specimen urine (CSU) is considered less susceptible to contamination. We compared MSU and CSU methods in term pregnancy to test these assumptions.

**Methods:**

Healthy pregnant women at term gestation (*n* = 32, median gestation 38 + 6 weeks, IQR 37 + 6–39 + 2) undergoing elective caesarean section provided a MSU and CSU for paired comparison that were each analysed for bacterial growth and bladder distress by fresh microscopy, sediment culture and immunofluorescent staining. Participants completed a detailed questionnaire on lower urinary tract symptoms. Epithelial cells found in urine were tested for urothelial origin by immunofluorescent staining of Uroplakin III (UP3), a urothelial cell surface glycoprotein. Urothelial cells with closely associated bacteria, or “clue cells”, were also counted. Wilcoxons signed rank test was used for paired analysis.

**Results:**

Women reported multiple lower urinary tract symptoms (median 3, IQR 0–8). MSU had higher white blood cell counts (median 67 vs 46, z = 2.75, *p* = 0.005) and epithelial cell counts (median 41 vs 22, z = 2.57, *p* = 0.009) on fresh microscopy. The proportion of UP3+ cells was not different (0.920 vs 0.935, z = 0.08, *p* = 0.95), however MSU had a higher proportion of clue cells (0.978 vs 0.772, z = 3.17, *p* = 0.001). MSU had more bacterial growth on sediment culture compared to CSU specimens (median 8088 total cfu/ml vs 0, z = 4.86, p = 0.001). Despite this, routine laboratory cultures reported a negative screening culture for 40.6% of MSU specimens.

**Conclusion:**

Our findings have implications for the correct interpretation of MSU findings in term pregnancy. We observed that MSU samples had greater bacterial growth and variety when compared to CSU samples. The majority of epithelial cells in both MSU and CSU samples were urothelial in origin, implying no difference in contamination. MSU samples had a higher proportion of clue cells to UP3+ cells, indicating a greater sensitivity to bacterial invasion. Urinary epithelial cells should not be disregarded as contamination, instead alerting us to underlying bacterial activity.

**Electronic supplementary material:**

The online version of this article (10.1186/s12884-017-1606-z) contains supplementary material, which is available to authorized users.

## Background

The urinary tract undergoes various physiological adaptations in pregnancy. The effect of increased progesterone leads to ureteral dilatation, reduced ureteral tone and an increased potential for glycosuria which encourages microbial growth [1, 2]. These changes predispose pregnant women to infections and 1–4% of women develop an acute bladder infection for the first time during pregnancy [3]. If untreated, bacteria may ascend the renal tract causing pyelonephritis, a major cause of maternal morbidity and mortality [4].

Current dogma defines a urinary tract infection (UTI) as the presence and multiplication of microorganisms in one or more structures of the urinary tract with associated tissue invasion [5]. This may present with symptoms, such as dysuria, frequency, urgency and suprapubic pain, or be asymptomatic. The routine urine culture techniques are designed to favour the growth of common uropathogens [6–9]. Threshold bacterial counts of ≥ 10^5^ total colony forming units per millilitre (cfu/ml) urine are used to discriminate between true infection and contamination of the urine sample by vulvo-vaginal organisms [10]. At present, this method of diagnosing UTIs is standard practice in UK laboratories, despite evidence of insensitivity [11, 12]. The majority of urine cultures do not surpass diagnostic thresholds in routine practice [13].

Contamination occurs when bacteria, from the skin surfaces, get into the urine culture. By proxy, a urine sample is presumed to be contaminated if epithelial cells are detected [10]. Historically, urethral catheterisation has been the gold standard for urine collection as it bypassed the skin surface and was thought to avoid skin contamination [14]. However, it has been found that voided urine samples may be equally valid to CSU samples, if not superior due to its convenience [15]. This non-invasive technique has since become the clean-catch, mid-stream urine (MSU) method which draws on the assumption that skin contamination is avoided by discarding the initial stream [16]. The MSU is now the most frequently used sampling method in today’s clinical setting [17, 18].

Recent evidence suggests that bacteriuria is more complex than previously thought. Healthy urine is no longer considered sterile; manifesting a microbiome that may include urinary pathogens [19–24]. Colonisation of the urothelium, by microbial biofilms adherent to cell surfaces or intracellular invasion, is now recognised to be important in the pathology of UTI [25–28]. The innate immune response to these events is to increase urothelial shedding so as to excrete parasitsed cells in the urine [28]. These “clue cells” are epithelial cells with closely associated bacteria and may be evidence of bladder infection, not skin contamination.

There is no single, validated method for urine sampling in pregnancy. It is unclear which technique is best for identifying true bacteriuria and discriminating against urine contamination in pregnant women [29]. The aim of this study was to compare MSU and CSU samples at term pregnancy for signs of bladder distress, bacterial infection and urine contamination, and to determine if CSU samples have a lower rate of contamination compared to MSU samples.

## Methods

This was a prospective, cross-sectional, single-blinded comparative study of two urinary sampling methods in pregnant women at term gestation undergoing elective caesarean section. Patients were recruited between June and August 2015 from the obstetric labour ward theatre at a tertiary hospital in central London and gave written consent to an ethically approved study. The data were structured so that individual patients could not be identified.

A set of 41 questions was used to obtain a pertinent clinical, obstetric and infection history. Further clinical data were gleaned from electronic patient notes and records. A lower urinary tract symptom (LUTS) profile was created using the “Artemis questionnaire”, a detailed 49 item questionnaire divided into four categories: stress incontinence symptoms, overactive bladder symptoms, voiding symptoms, and pain symptoms (Additional file 1). This questionnaire is used in clinical practice by a tertiary chronic LUTS clinic as a tool to identify urine infections and to assess treatment response in non-pregnant patients with chronic LUT symptoms [30–33]. In this study, the LUTS profile was used as an assessment of bladder distress.

Two urine samples were obtained from each participant for paired comparison. First, participants were instructed to provide a MSU sample before entering the labour ward operating theatre. Verbal and written instructions were given to ensure an adequate clean-catch sample by (1) washing hands and cleaning the genital area with a hypoallergenic wipe to prevent local contamination; (2) parting the labia and urinating the first part of the stream into the toilet before moving the sterile container into the urine stream; and (3) removing the container before urination was complete. After completion of the epidural and/or spinal anaesthesia, and prior to routine prophylactic antibiotic administration and caesarean section surgery, a transurethral urinary catheter was placed. The first 25 ml of urine was collected as the CSU sample.

### Fresh urine analysis

Each specimen was processed fresh and unspun at the same facility as sample collection within one hour in order to prevent cell degradation [32]. Urinary dipstick results for nitrites and leukocyte esterase, by-products of bacteria and leukocytes respectively, were recorded as evidence of bacterial infection. Urinary adenosine-5′-triphosphate (ATP), an inflammatory cytokine, was assayed by a luciferin-luciferase method in order to quantify bladder distress [34, 35]. Microscopy was performed using a haemocytometer chamber to quantify erythrocytes (red blood cells, RBC), leukocytes (white blood cells, WCC), and epithelial cells (EPC) as previously described by Horsley et al. [28].

### Immunofluorescent staining

Uroplakin III (UP3), an asymmetric glycoprotein expressed solely in the cell membrane of urothelial cells, has been shown to be a specific marker of terminal urothelial differentiation [36, 37]. We targeted UP3 through immunofluorescence in order to identify urothelial cells originating from the urinary tract. 4″, 6-diamidino-2-phenoylindole (DAPI), a nucleic acid counterstain, was used to identify genetic material from urothelial cells and bacteria. An immunofluorescence technique derived from Horsley et al. [28] was used to discriminate exfoliated urothelial cells (bladder distress, UP3+ and DAP+) from vulvo-vaginal epithelial cells (contamination, UP3- and DAP+). Contamination was measured by the ratio of epithelial cells to urothelial cells. Urothelial cells with closely associated bacteria (clue cells, UP3+ and DAPI+ host and bacteria) were identified as evidence of bladder urothelium shedding in response to infection, and bladder distress was measured by the proportion of clue cells to urothelial cells. An epi-fluorescent microscopy was used at ×40 and ×100 oil magnification and counts were performed in triplicate to quantify UP3+ cells, DAPI+ cells, and clue cells. Images were processed and analysed using Infinity Capture and Analyze V6.2.0 and Image J 1.46r software.

### Sediment culture

Individual bacteria species were identified using a technique previously described by Khasriya et al. [21]. Urine was centrifuged and the resulting sediment resuspended and cultured on chromogenic CPS3 culture media (bioMérieux). The plates were incubated in aerobic conditions at 37-degree C for 24–48 h and bacterial colonies were identified and quantified. Bacterial isolates were characterised by genus, and where possible species, using the following phenotypic features: key characteristics on chromogenic agar, morphology for cocci and bacilli on microscopy, Gram staining, and biochemical tests for indole, oxidase and catalase reactions. Full species identification by molecular testing was not performed as it was beyond the scope of this study. A small portion of the sample was sent for routine culture at an accredited clinical laboratory using standard methods [13] in order to permit comparison of study culture methods to routine culture methods.

### Statistics

The sample size (α = 0.05, 1-β = 0.80, *n* = 25) was calculated using SPSS SamplePower and statistical analysis was conducted using SPSS Statistics 22 (IBM, New York, USA). Wilcoxon signed rank test was used for paired analysis of two related non-parametric variables, where the absolute difference between each pair was ranked and the summation of mean positive and negative ranks generated as a z score. A two-sided exact *p* value ≤0.05 was considered statistically significant. Where appropriate, linear correlation was expressed as Pearson’s coefficient (r) and a significant score defined as an exact, two-tailed p value ≤0.05. Samples were analysed blind to their collection method.

## Results

### Study profile

Between 30 June and 13 August 2015, 32 pregnant women attending for elective caesarean section consented to participate in the study. A MSU and CSU sample was collected from each participant, with a median time of 2.5 h between collection of samples (IQR 1.5–3.0). No participants were lost to follow-up, and paired analysis of MSU and CSU data was performed for all women.

The median age was 35 years (range 23 to 44). The most common ethnicity for the patient and her partner was ‘White’, and most women were non-smokers with a normal or overweight pre-pregnancy BMI. The median gestation was 38 + 6 weeks, with 39 weeks being the most frequent gestation (18.8%). Most women were multiparous (81.2%) (Table 1). The commonest primary reason for elective caesarean section was history of a previous caesarean section (40.6%). All deliveries resulted in a live birth, and there were five admissions to the neonatal unit. Two infants were anticipated admissions due to previously known congenital defects. Three infants were febrile in the immediate postnatal period, but no infectious source was identified despite extensive investigation. There was one neonatal death within 24 h of life, however multiple anatomical anomalies had been noted on antenatal ultrasound scans and the infant had not been expected to survive.

**Table 1:** Selected obstetric characteristics

Obstetric characteristics		
Gestation in weeks	38 + 6	(37 + 6–39 + 2)
Primiparous	6	(18.8)
Multiparous	26	(81.2)
Primary indication for elective caesarean section		
Previous caesarean section	13	(40.6)
Breech presentation	6	(18.8)
Placenta praevia	2	(6.3)
Twin pregnancy	1	(3.1)
Previous traumatic delivery	3	(9.4)
Medical co-morbidity	6	(18.8)
Maternal choice	1	(3.1)
Infant		
Live births	32	(100)
Birthweight in grams	3.225	(2900–3635)
Admission to neonatal unit	5	(15.6)

Data reported as n (%) or median (IQR)

### Lower urinary tract symptoms

The frequency of daytime voids varied from three to over 20 times per day (Table 2). Incontinence, daytime or nocturnal, was not a feature for most women. Five women (15.2%) reported using a pad for leakage, and no participants required intermittent or permanent indwelling catheterisation. Symptoms of overactive bladder were the most commonly reported symptoms, and most women had a total count of three LUTS (IQR 0–8). Most women who reported symptoms stated they had occurred in recent months (69.7%), while a small proportion of women (12.1%) reported symptoms of a more chronic duration of years.

**Table 2:** Summary of LUTS identified using the Artemis questionnaire

Lower urinary tract symptoms		
Daytime frequency	9	(6–12)
Nocturnal frequency	2	(1–4)
Daytime incontinence	0	(0- > 5/d)
Nocturnal incontinence	0	(0–0)
Pad incontinence	5	(15.2)
Incontinence requiring catheterisation	0	(0–0)
Stress incontinence symptoms	0	(0–1.8)
Overactive bladder symptoms	1	(0–3)
Voiding symptoms	0	(0–2)
Pain symptoms	0	(0–0)
Total symptoms	3	(0–8)
Duration of symptoms		
Antenatal period only	23	(69.7)
Pre-pregnancy	4	(12.1)
Not answered	6	(18.2)

Data reported as *n* (%) or median (IQR)

### Antenatal history of urinary tract infection

All 32 participants had a MSU culture at their first antenatal booking appointment, and no women required antibiotic therapy based on their booking sample. In 14 booking samples (43.8%), there was evidence of bacterial growth that did not reach the routine laboratory threshold (total cfu/ml ≥ 10^5^) for treatment. The remaining 18 booking samples were reported as negative by the laboratory. As routine antenatal care requires only one MSU culture at booking, any repeat cultures during the subsequent antenatal period were performed on clinical suspicion of UTI. Ten participants had repeat MSU cultures, three of which met diagnostic criteria for UTI; two women had persistent, but asymptomatic, growth of group B Streptococcus despite treatment, and one woman had a symptomatic Enterococcus UTI which was successfully treated with oral antibiotics. No participants developed sepsis throughout their antenatal, intra-partum and post-partum periods.

### Fresh urine analysis

Each sample was assessed as a fresh unspun specimen using urinary dipstick, ATP concentration assay and microscopy for cell count (Table 3) and then compared by paired analysis. MSU samples were of larger volume than CSU samples (75 ml vs 30 ml, z = 4.45, *p* = 0.001), but had no difference in specific gravity thereby demonstrating similar volumetric concentrations for both sample types. MSU samples were more likely to contain leukocyte esterase (z = 4.61, p = 0.001). There was no difference in protein concentration, and all specimens tested negative for nitrites. Compared to MSU samples, CSU samples contained higher concentrations of ATP (2504 vs 12 attamoles, z = 4.56, p = 0.001) and higher proportions of RBC (7 vs 4 per 1 μl, z = 2.786, *p* = 0.004). WBC counts and EPC counts were higher in MSU specimens compared to CSU samples (67 vs 46 per 1 μl, z = 2.75, *p* = 0.005 and 41 vs 22 per 1 μl, z = 2.57, *p* = 0.009 respectively).

**Table 3:** Fresh specimen results and paired analysis

Variable	MSU *n* = 32		CSU *n* = 32		Z	*P*
Volume in millilitres	75	(41.25–100)	30	(25–40)	−4.45	<0.001*
Specific gravity	1.010	(1.005–1.015)	1.010	(1.010–1.015)	0.00	1.00
Protein						
Negative	17	(53.1)	18	(56.3)		
Trace	10	(31.3)	9	(28.1)	0.00	1.00
+	4	(12.5)	3	(9.4)		
++	1	(3.1)	2	(6.3)		
Leukocyte esterase						
Negative	4	(12.5)	19	(59.4)	−4.61	<0.001*
Trace	6	(18.8)	11	(34.4)		
+	12	(37.5)	2	(6.3)		
++	2	(6.3)	0	–		
+++	8	(25.0)	0	–		
ATP concentration^a^	2504	(1415–5779)	12,323	(8250–2098)	−4.56	<0.001*
RBC count^b^	4	(0.5–12.0)	7	(2.0–36.0)	−2.79	0.004*
WBC count^b^	67	(40.5–135.5)	46	(32.0–70.5)	−2.75	0.005*
EPC count^b^	41	(17.5–69.5)	22	(10.5–36.5)	−2.57	0.009*

Data reported as *n* (%) or median (IQR) and paired analysis performed by Wilcoxon signed ranks test. MSU samples had significantly higher leukocyte esterase, ATP concentration, and cell counts

^a^Concentration described in attamoles, or mole × 10^−18^

^b^Red blood cell (RBC), white blood cell (WCC), epithelial cell (EPC) per high power field

### Immunofluorescent staining

**Fig. 1:**
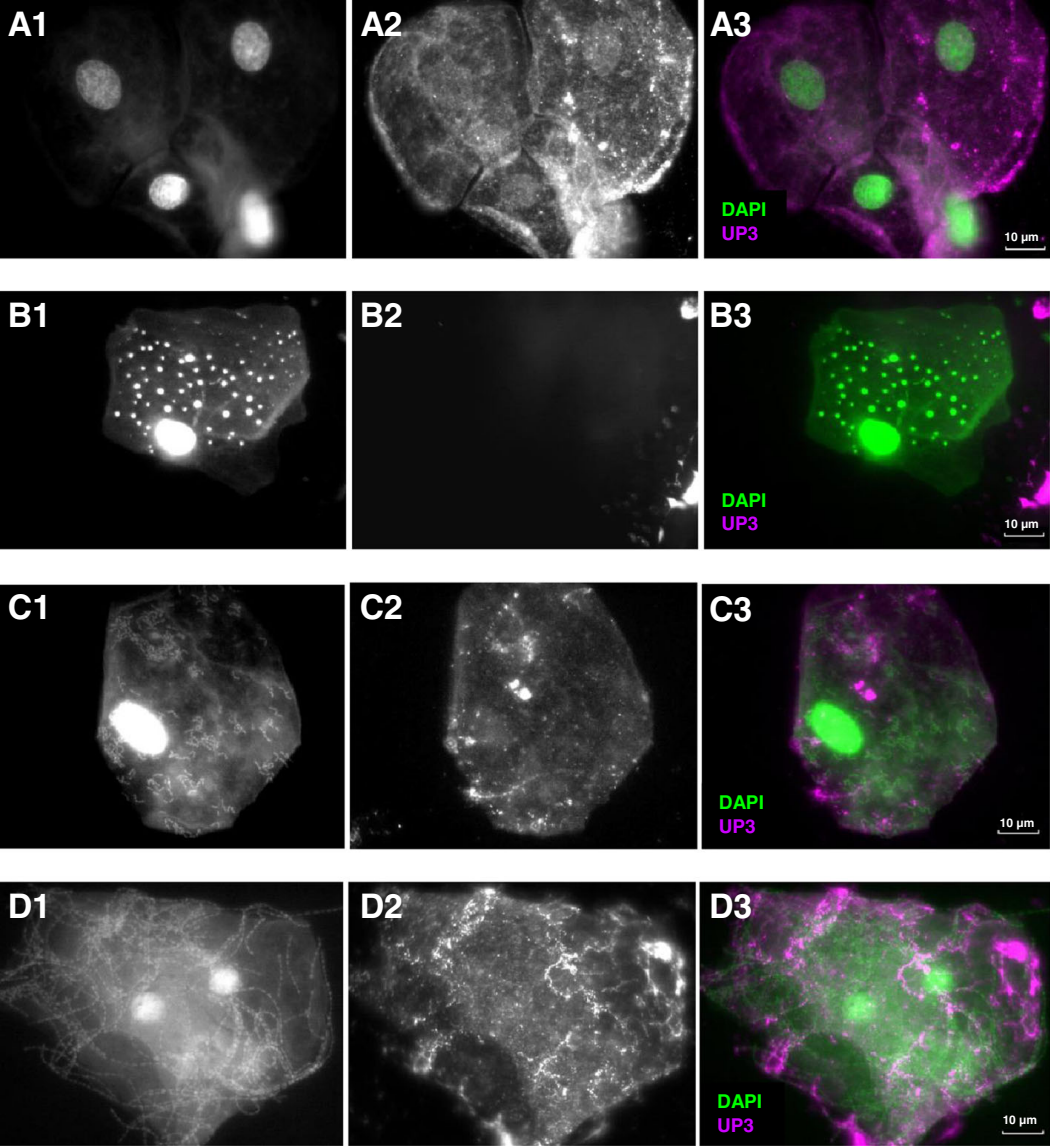
Immunofluorescence under epi-fluorescent microscopy. Description: 4″, 6-diamidino-2-phenoylindole (DAPI) and Uroplakin-III (UP3) stain for DNA and terminally differentiated urothelial surface membrane glycoprotein respectively. Each channel for DAPI (1) and UP3 (2) shown separately in monochrome, then merged as a composite (3) showing the expression of DAPI in green and UP3 in magenta. All images viewed at ×100 magnification with the white scale bar sized at 10 μm. **a**) CSU specimen of UP3+ bladder epithelial cells. **b**) MSU specimen of UP3- vulvo-vaginal epithelial cell with associated inclusion bodies. **c**) Single UP3+ clue cell with associated bacteria. **d**) Two overlapping UP3+ clue cells with associated bacteria

**Fig. 2:**
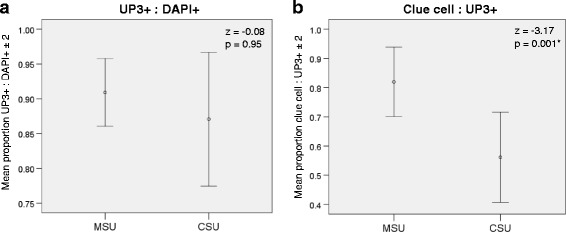
Cell proportions. Description: Graph showing median cell proportions in MSU samples when compared to CSU samples. **a**) There is a non-significantly lower proportion of UP3+ to DAPI+ cells in CSU samples compared to MSU samples (z = 0.08, p = 0.95). **b**) There is a significantly lower proportion of clue cells to UP3+ cells in CSU specimens compared to MSU specimens (z = 3.17, *p* = 0.001)

DAPI+ cells with a positive UP3 signal were identified as cells originating from terminally differentiated urothelium (Fig. 1a). Cells that only stained DAPI+ were considered to originate from the vulvo-vaginal skin (Fig. 1b). Clue cells were shown as urothelial cells with closely associated bacteria, as evidenced by UP3+ cells with additional non-nuclear DAPI+ signal (Fig. 1c and d). Proportions were calculated comparing different cell types. There was no significant difference in the proportion of UP3+ cells to DAPI+ cells (median 0.920, IQR 0.820–0.995 vs 0.935, IQR 0.833–1.000, z = 0.08, *p* = 0.95). There was a higher proportion of clue cells to UP3+ cells in MSU compared to CSU samples (median 0.978, IQR 0.865–1.000 vs 0.772, IQR 0.033–0.978, z = 3.17, *p* = 0.001). Graphs have been used to further illustrate the median and standard error means for UP3+ to DAPI+ (Fig. 2a) and clue cells to UP3+ proportions (Fig. 2b).

### Sediment culture

One MSU sample was not received by the hospital laboratory, and the paired sample was excluded from sediment culture and routine laboratory culture. On sediment culture, MSU samples had a far greater total cfu/ml than CSU samples (median 8088, IQR 1239–76,064 vs median 0, IQR 0–38, z = 4.86, *p* = 0.001). There was a higher number of species grown per specimen in MSU compared to CSU samples (z = 4.78, *p* = 0.001) (Table 4). The majority of MSU specimens had a mixed growth of ≥2 bacteria (88.3%) while most CSU samples had no growth (47.1%) or growth of only one bacterial species (35.3%). In routine laboratory cultures, most MSU and CSU samples reported a negative screening culture (40.6% and 87.5% respectively). The sediment culture was more likely to report bacterial growth than routine laboratory culture (z = 3.66, *p* = 0.001).

**Table 4:** Bacterial culture and paired analysis

Variable	MSU *n* = 31		CSU *n* = 31		Z	*P*
Number of species identified						
No growth	0	–	16	(47.1)		
1	2	(5.9)	12	(35.3)		
2	6	(17.6)	3	(8.8)	−4.78	<0.001*
3	11	(32.4)	1	(2.9)		
4	11	(32.4)	0	–		
5	2	(5.9)	0	–		
Routine laboratory culture^a^						
Screening culture negative	13	(40.6)	28	(87.5)	−3.66	<0.001*
No significant growth	6	(18.8)	1	(3.1)		
Mixed growth, uncertain significance	3	(9.4)	0	–		
Growth × 10^4^–10^5^	1	(3.1)	0	–		
Growth × 10^5^	8	(25.0)	0	–		
Growth, no bacterial count	0	–	2	(6.3)		
Missing	1	(3.1)	1	(3.1)		

Data reported as n (%) or median (IQR) and paired analysis performed by Wilcoxon signed ranks test. MSU samples grew more bacteria on sediment culture and routine laboratory culture

^a^Treatment threshold by routine laboratory culture set at total cfu/ml ≥ 10^5^

MSU specimens grew more bacterial types (total isolates 103 vs. 19) with Staphylococcus as the commonest isolated genus (37, 35.9%) (Table 5). CSU specimens had less bacterial growth and variety. Four colonies (MSU = 2, 1.9% and CSU = 2, 10.5%) were seen on chromogenic agar following aerobic incubation, but the genus could not be identified despite biochemical testing and repeat subculture.

**Table 5:** Frequency of bacterial species isolated

Species	MSU *n* (%)	CSU *n* (%)
Staphylococcus	37 (35.9)	4 (21.1)
Enterococcus	20 (19.4)	2 (10.5)
*Escherichia coli*	17 (16.5)	0 (0.0)
Lactobacillus	9 (8.7)	4 (21.1)
Streptococcus	9 (8.7)	3 (15.8)
Corynebacterium	5 (4.9)	2 (10.5)
Yeast	3 (2.9)	2 (10.5)
Proteus	1 (1.0)	0 (0.0)
Unidentifiable^a^	2 (1.9)	2 (10.5)
Total number of species	103	19

^a^Unidentified bacteria were colonies that could not be isolated and were recultured in aerobic conditions on chromogenic culture media or chocolate blood agar, and therefore it was not possible to perform gram stain or biochemical tests for identification

Total cfu/ml on sediment culture for both MSU and CSU samples were tested for evidence of correlation with three EPC variables: EPC count on fresh microscopy, proportion of UP3+ to DAPI+ cells, and clue cells to UP3+ cells (Table 6). There was no significant correlation, irrespective of sampling method.

**Table 6:** Correlation between total cfu/ml and EPC variables

Total cfu/ml	EPC count		UP3+: DAPI+		Clue cells: UP3+	
	R	P	R	P	R	P
MSU	0.13	0.47	−0.15	0.43	0.16	0.39
CSU	0.005	0.98	0.15	0.41	0.19	0.31

Linear correlation expressed as Pearson’s coefficient (r). There was no significant correlation between total cfu/ml and EPC variables for both MSU and CSU samples

## Discussion

In this study of pregnant women at term, we observed that MSU samples had greater bacterial growth and variety when compared to CSU samples. The majority of epithelial cells in both MSU and CSU samples were urothelial in origin, implying no difference in contamination rates. Furthermore, MSU samples had a higher proportion of clue cells to UP3+ cells, and may indicate a greater degree of bacterial invasion when compared to urine sampled directly from the bladder. This has implications for the correct interpretation of MSU findings in late pregnancy.

UK laboratories diagnose urinary tract infections using a threshold of ≥10^5^ total cfu/ml first described in 1957 by Kass et al. [10]. This standard persists despite having been shown to misdiagnose over 50% of women with lower urinary tract symptoms [14]. A much lower count of ≥10^2^ cfu/ml of a single isolate has been suggested as a more appropriate threshold for infection in acutely symptomatic women [11, 38]. In the event of mixed bacterial growth, the sample is considered to be contaminated by vulvo-vaginal bacterial flora [39, 40]. Within our term pregnant population, the majority of MSU sample sediment cultures had mixed growth and total cfu/ml > 10^3^. Our sediment culture appears to correlate with clinical presentation as most participants reported multiple lower urinary tract symptoms. However, they were classified as “no significant growth” or “mixed growth of uncertain significance” by routine laboratory culture. It is possible also that symptomatic pregnant women of term gestation with mixed growth and fewer bacterial colonies may not be appropriately treated when standard means are used.

The uroplakin analysis refutes the assumption that epithelial cells are vulvo-vaginal skin contaminants. The epithelial cells seen in the urine were urothelial in origin and were often clue cells with closely associated bacteria. MSU samples had more clue cells and therefore a greater degree of bacterial invasion. There was no significant correlation between total cfu/ml and clue cells, but this is to be expected as intracellular bacteria will evade detection by routine culture means. Our findings confirm that colonisation of urothelial cells by microbes leads to an innate immune response as previously described by Horsley and colleagues [28]. Mucosal surfaces are a common entry point for microbes, and in the context of urinary tract infections, uropathogenic bacteria invade the apical surface of the urothelium to create adherent surface biofilms or form intracellular bacterial colonies. The parasitised urothelial cell is subsequently exfoliated into the urine to encourage bacterial clearance [21, 27, 28, 41, 42], leading to the accumulation of these clue cells as sediment at the base of the bladder. While a transurethral catheter will pass through this deposit and sample the urine above, a MSU is more likely to achieve a greater sample of clue cells. It is conceivable that an ordinary void may achieve even more by including the first part of the stream.

Urine is increasingly recognised to have its own diverse microbiome, and standard culture methods only capture a small fraction of this living community [19, 22–25]. Imbalances within this healthy microbiota are associated with factors such as age, body mass index and hormonal status and predisposition to disease [20]. Pregnancy may be a similarly influential factor. Current quantitative criteria may be inappropriate for diagnosing infection given the true urinary microbiome. Sediment culture delivers more microbes, but the pathological significance of these organisms is unexplained. We propose that the appropriate substrate for analysis of infection should be urothelial cells as they have been parasitised by the pathogen. Khasriya et al. [21] provided evidence supporting this contention outside pregnancy. Intracellular uropathogens may evade detection by standard diagnostic means. This degree of urothelial parasitisation in voided urine implies that the MSU may be superior in capturing the disease signals.

## Strengths and limitations

The strength of our study is the comparative design in which participants provide their own control thereby eliminating sampling confounders. Another strength was an effective use of immunofluorescent staining to refute the claim of contamination and, through the identification of clue cells, demonstrate bladder immune response to cellular invasion by bacteria. However, as UP3 is expressed in all terminally differentiated urothelial cells including both the bladder and urethra, we were unable to ascertain the precise origin of UP3+ cells. Another limitation of our study was that bacteria were identified to the genus and the species was not always identifiable. This is particularly relevant in the case of Staphylococcus sp., a bacterium frequently cultured in MSU samples. The isolated colonies may be *S. saprophyticus*, the second most common causative organism of UTIs in this demographic, or *S. epidermis* and *S. aureus*, species typically found on skin surfaces. Further identification of bacteria through molecular means is currently underway to clarify our sediment culture. Finally, it is unclear whether our high urothelial cell counts were wholly due to infection or as a result of physiological hormonal changes. In vitro work with human urothelial cells has demonstrated progesterone to induce cell proliferation leading to increased cell turnover and exfoliation [43]. Further work is required to characterise bladder urothelial behaviour throughout the hormonal changes of pregnancy.

## Conclusion

In this study of pregnant women at term, we observed that MSU samples had greater bacterial growth and variety when compared to CSU samples. The majority of epithelial cells in both MSU and CSU samples were urothelial in origin, suggesting no difference in contamination rates. Furthermore, MSU samples had a higher proportion of clue cells to UP3+ cells, and may indicate a greater propensity to detect bacterial invasion compared to CSU sampling. A closer examination of the MSU for clue cells may prove to be more informative on disease state from current practice. This has implications for the correct interpretation of MSU findings in late pregnancy.

## Additional file

Additional file 1: Artemis questionnaire. Description: Detailed 49 item questionnaire divided into four categories: stress incontinence symptoms, overactive bladder symptoms, voiding symptoms, and pain symptoms used to create a lower urinary tract symptom (LUTS) profile as an assessment of bladder distress. (DOCX 145 kb)

## Abbreviations

ATP: Adenosine-5′-triphosphate
cfu/ml: Colony forming units per millilitre
CSU: Catheter specimen of urine
DAPI: 4″, 6-diamidino-2-phenoylindole
EPC: Epithelial cell
LUTS: Lower urinary tract symptom(s)
MSU: Midstream urine
RBC: Erythrocyte (red blood cell)
UK: United Kingdom
UP3: Uroplakin III
UTI: Urinary tract infection
WBC: Leukocyte (white blood cell)

## References

[1] Patterson TF, Audriole VT (1987). Bacteriuria in pregnancy. Infect Dis Clin N Am.

[2] Mikhail MS, Anyaegbunam A (1995). Lower urinary tract dysfunction in pregnancy: a review. Obstet Gynecol Surv.

[3] Foxman B (2002). Epidemiology of urinary tract infections: incidence, morbidity and economic costs. Am J Med.

[4] Farkash E, Weintraub AY, Sergienko R, Wiznitzer A, Zlotnik A, Sheiner E (2012). Acute antepartum pyelonephritis in pregnancy: a critical analysis of risk factors and outcomes. European journal of obstetrics and Gynaecology and. Reprod Biol.

[5] Drekonja DM, Johnson JR (2008). Urinary tract infections. Primary Care.

[6] Ronald A (2002). The etiology of urinary tract infection: traditional and emerging pathogens. Am J Med.

[7] Karlowsky JA, Kelly LJ, Thornsberry C, Jones ME, Sahm DF (2002). Trends in antimicrobial resistance among urinary tract infection isolates in Escherichia Coli from female outpatients in the United States. American Society of Microbiology.

[8] Wallmark G, Arremark I, Telander B (1978). Staphylococcus saprophyticus: a frequent cause of acute urinary tract infection among female outpatients. J Infect Dis.

[9] Eisenstadt J, Washington JA, Mobley HLT, Warren JW (1996). Diagnostic microbiology for bacteria and yeasts causing urinary tract infection. Urinary tract infections – molecular pathogenesis and clinical management.

[10] Kass EH (1957). Bacteriuria and the diagnosis of infection in the urinary tract. Arch Intern Med.

[11] Stamm WE, Counts GW, Running KR, Fihn S, Turck M, Holmes KK (1982). Diagnosis of coliform infection in acutely dysuric women. New England Journal of Medicine.

[12] Gupta K, Hootan TM, Stamm WE (2001). Increasing antimicrobial resistance and the management of uncomplicated community-acquired urinary tract infections. Ann Intern Med.

[13] Unit S, Services M. UK standards for microbiology investigations: investigation of urine (bacteriology 41). Public Health England. 2014;7(2)

[14] Boshell BR, Sanford JPA (1957). Screening method for the evaluation of urinary tract infections in female patients without catheterisation. Ann Intern Med.

[15] Beeson PB (1958). The case against the catheter. Am J Med.

[16] Clinical and Laboratory Standards Institute (CLSI). Urinalysis; Approved Guideline – Third Edition. CLSI document GP16-A3 (ISBN 1–56238–687-5). Clinical and Laboratory Standards Institute, 950 West Valley Road, Suite 2500, Wayne, Pennsylvania 19087 USA, 2009.

[17] Walter FG, Knopp RK (1989). Urine sampling in ambulatory women: midstream clean-catch versus catheterisation. Ann Emerg Med.

[18] Ware TT, Jones SR, Reese RE, Betts RF (1996). Genitourinary tract infections. A practical approach to infectious diseases.

[19] Finucane TE (2017). ‘Urinary tract infection’ and the microbiome. AJM.

[20] Thomas-White KJ, Kliethermes S, Rickey L (2017). Evaluation of the urinary microbiota of women with uncomplicated stress urinary incontinence. AJOG.

[21] Khasriya R, Sathiananthamoorthy S, Ismail S, Kelsey M, Wilson M, Rohn JL, Malone-Lee J (2013). Spectrum of bacterial colonisation associated with urothelial cells from patients with chronic lower urinary tract symptoms. J Clin Microbiol.

[22] Wolfe AJ, Toh E, Shibata N, Rong R, Kenton K, Fitzgerald M, Mueller ER, Schreckenberger P, Dong Q, Nelson DE, Brubaker L (2012). Evidence of uncultivated bacteria in the adult female bladder. J Clin Microbiol.

[23] Fouts DE, Pieper R, Szpakowski S, Pohl H, Knoblach S, Suh MJ, Huang ST, Liungberg I, Sprague BM, Lucas SK, Torralba M, Nelson KE, Groah SL (2012). Integrated net-generation sequencing of the 16S rDNA and metaproteomics differentiate the healthy urine microbiome from asymptomatic bacteriuria in neuropathic bladder associated with spinal cord injury. J Transl Med.

[24] Hilt EE, McKinley K, Pearce MM, Rosenfeld AB, Zilliox MJ, Mueller ER, Brubaker L, Gai X, Wolfe AJ, Schreckenberger PC (2014). Urine is not sterile: use of enhanced urine culture techniques to detect resident bacterial flora in the adult female bladder. J Clin Microbiol.

[25] Apodaca G (2004). The uroepithelium: not just a passive barrier. Traffic.

[26] Birder L, Andersson KE (2013). Urothelial signalling. Physiology Review.

[27] Anderson GG, Palermo JJ, Schilling JD, Roth R, Heuser J, Hultgren SJ (2003). Intracellular bacterial biofilm-like pods in urinary tract infections. Science.

[28] Horsley H, Malone-Lee J, Holland D, Tuz M, Hibbert A, Kelsey M, Kupelian A, Rohn JL (2013). Enterococcus faecalis subverts and invades the host urothelium in patients with chronic urinary tract infection. PLoS One.

[29] Schneeberger C, van den Heuvel ER, Erwich JHM, Stolk RP, Visser CE, Geerlings SE (2013). Contamination rates of three urine-sampling methods to assess bacteriuria in pregnant women. Obstetrics & Gynecology.

[30] Ghei M, Malone-Lee J (2005). Using the circumstances of symptom experience to assess the severity of urgency in the overactive bladder. J Urol.

[31] Al-Buheissi S, Khasriya R, Maraj BH, Malone-Lee JA (2008). Simple validated scale to measure urgency. J Urol.

[32] Kupelian A, Horsley H, Khasriya R, Amussah R, Badiani R, Courtney AM, Chandhyoke NS, Riaz U, Savlani K, Moledina M, Montes S, O’Conner D, Visavadia R, Kelsey M, Rohn JL, Malone-Lee J (2013). Discrediting microscopic pyuria and lueocycte esterase as diagnostic surrogates for infection in patients with lower urinary tract symptoms: results from a clinical and laboratory evaluation. British Journal of Urology International.

[33] Khasriya R, De Iorio M, Barcella W, Malone-Lee J (2016). A measure of the symptoms of chronic urinary tract infection in women that captures treatment effects (for submission).

[34] Van der Wijk T, De Jonge HR, Tilly BC. Osmotic cell swelling-induced ATP release mediates the activation of extracellular signal-regulated protein kinase (Erk)-1/2 but not the activation of osmo-sensitive anion channels. J Biochem 1999;343(Pt 3):579–586. PubMed PMID: 10527936. Pubmed Central PMCID: 1220589. Epub 1999/10/21. eng. 10.1042/0264-6021:3430579 PMC1220589 10527936

[35] Gill K, Horsley H, Kupelian A, Baio G, de Iorio M, Sathiananamoorthy S, Khasriya R, Rohn JL, Wildman SS, Malone-Lee J, Urinary ATP. As an indicator of infection and inflammation of the urinary tract in patients with lower urinary tract symptoms. BMC Urol. 2015;15(7) doi:10.1186/s12894-015-0001-1. 10.1186/s12894-015-0001-1 PMC4351839 25886951

[36] XR W, Sun TT (1993). Molecular cloning of a 47 kDa tissue-specific and differentiation-dependent urothelial cell surface glycoprotein. J Cell Sci.

[37] XR W, Lin JH, Walz T, Haner M, Yu J, Aebi U, Sun TT (1994). Mammalian uroplakins: a group of highly conserved urothelial differentiation-related membrane proteins. J Biol Chem.

[38] Kunin CM, White LV, Hua THA (1993). Reassessment of the importance of "low-count" bacteriuria in young women with acute urinary symptoms. Ann Intern Med.

[39] Graham JC, Galloway AACP (2001). Best practice no. 167: the laboratory diagnosis of urinary tract infection. J Clin Pathol.

[40] McCarter YS, Burd EM, Hall GS, Zervos M (2009). Cumitech 2C, laboratory diagnosis of urinary tract infections. Sharp SE (Ed). 2009.

[41] Rosen DA, Hooton TM, Stam WE, Humphrey PA, Hultgren SJ (2007). Detection of intracellular bacterial communities in human urinary tract infection. PLoS Med.

[42] Thumbikat P, Berry RE, Zhou G, Billips BK, Yaggie RE, Zaichuk T, Sun TT, Schaeffer AJ, Klumpp DJ (2009). Bacteria-induced uroplakin signalling mediates bladder response to infection. PLoS Pathog.

[43] Teng J, Wang ZY, Bjorling DE (2003). Progesterone induces the proliferation of urothelial cells in an epidermal growth factor dependent manner. J Urol.

