# Supplementary material for: Urothelial cells may indicate underlying bacteriuria in pregnancy at term: a comparative study
Source: BMC Pregnancy Childbirth. 2017 Dec 8;17:414. doi: 10.1186/s12884-017-1606-z (PMC5723065; doi:10.1186/s12884-017-1606-z)
Supplement: Additional file 1: — Artemis questionnaire. Description: Detailed 49 item questionnaire divided into four categories: stress incontinence symptoms, overactive bladder symptoms, voiding symptoms, and pain symptoms used to create a lower urinary tract symptom (LUTS) profile as an assessment of bladder distress. (DOCX 145 kb) [file 12884_2017_1606_MOESM1_ESM.docx]

# Appendix 1: Artemis Quetionnaire

ARTEMIS HISTORY FORM

**Indicate** (X) how likely or unlikely you feel like you **might have a urinary tract infection TODAY**


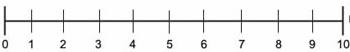


Definitely have a UTI today

Definitely do not have UTI today

Please **circle** which answer most describes your urinary symptoms throughout **THIS** pregnancy.

|  | | | | | | |
| --- | --- | --- | --- | --- | --- | --- |
| **Daytime Frequency**  *How often do you pass urine during the day?* | 1-2 | 5-6 | 9-10 | 13-14 | | 17-18 |
|  | 2-3 | 6-7 | 10-11 | 14-15 | | 18-19 |
|  | 3-4 | 7-8 | 11-12 | 15-16 | | 19-20 |
|  | 4-5 | 8-9 | 12-13 | 16-17 | | >20 |
|  | | | | | | |
| **Nocturia**  *How often do you get up to pass urine at night?* | 0 | 1-2 | 3 | 4-5 | | 6 |
|  | 0-1 | 2 | 3-4 | 5 | | >6 |
|  | 1 | 2-3 | 4 | 5-6 | |  |
|  | | | | | | |
| **Daytime Incontinence**  *How often do you leak urine during the day?*  *D = day, W = week, M = month, Y = year*  *PMD = post-micturition dribbling* | 0 | 0-2D | 1-3D | 2-4D | | 4-5D |
|  | 0-1D | 1-2D | 2-3D | 3-4D | | >5D |
|  | 1-2W | 1-3W | 2-3W | 4-5W | | 4-6W |
|  | 1-2M | 1-3M | 1-4M | 1-2Y | | 1-3Y |
|  | 3-4Y | 3-5Y | 3-6Y | PMD | |  |
|  | | | | | | |
| **Nocturnal Incontinence**  *How often do you leak urine at night?*  *D = day, W = week, M = month, Y = year*  *PMD = post-micturition dribbling* | 0 | 0-2N | 1-3N | 2-4N | | 4-5N |
|  | 0-1N | 1-2N | 2-3N | 3-4N | | >5N |
|  | 1-2W | 1-3W | 2-3W | 4-5W | | 4-6W |
|  | 1-2M | 1-3M | 1-4M | 1-2Y | | 1-3Y |
|  | 3-4Y | 3-5Y | 3-6Y | PMD | |  |
|  | | | | | | |
| Duration of symptoms | | | | | Years | Months |
| Pad dependent incontinence – do you need to wear pads to prevent leakage? | | | | | Yes | No |
| CISC – do you perform Clean Intermittent Self Catheterisation? | | | | | Yes | No |
| Permanent indwelling catheter – do you have/have had a permanent urinary catheter? | | | | | Yes | No |
|  | | | | | | |
| **Stress incontinence symptoms** | | | | **Some** | | **None** |
| Cough/Sneeze incontinence – do you leak when you cough or sneeze? | | | | Yes | | No |
| Exercise incontinence – do you leak when you exercise? | | | | Yes | | No |
| Lifting incontinence – do you leak when you lift something? | | | | Yes | | No |
| Laughing incontinence – do you leak when you laugh? | | | | Yes | | No |
| Standing incontinence – do you leak on standing up? | | | | Yes | | No |
| Positional incontinence – do you leak when you change positions? | | | | Yes | | No |
| Passive incontinence – do you leak with no reason/without the feeling you want to go? | | | | Yes | | No |
|  | | | | | | |

|  | | |
| --- | --- | --- |
| **Overactive bladder symptoms** | **Some** | **None** |
| Urinary urgency – do you have a sudden need to rush to the toilet to urinate? | Yes | No |
| Urinary urge incontinence – when rushing to the toilet, do you leak before getting there? | Yes | No |
| Cold weather exacerbation – does cold weather make your urgency worse? | Yes | No |
| Running water urgency – does the sound of running water make your urgency worse? | Yes | No |
| Running water incontinence – have you leaked urine on hearing running water? | Yes | No |
| Latchkey urgency – do you need to pass urine when you put the key in your front door? | Yes | No |
| Latchkey incontinence – do you leak urine when you put the key in your front door? | Yes | No |
| Waking rising urgency – do you have to rush to the toilet on waking up? | Yes | No |
| Waking rising incontinence – do you leak urine on waking up? | Yes | No |
| Anxiety fatigue aggravation – does your urgency worsen when you are tired or anxious? | Yes | No |
| Premenstrual aggravation – does your urgency worsen prior to a period? | Yes | No |
| Leaking when coughing with urgency – if there’s urgency and coughed, would you leak? | Yes | No |
|  | | |
| **Voiding symptoms** | **Some** | **None** |
| Hesitancy – is there delay before you start to urinate? | Yes | No |
| Reduced stream – do you feel the urine stream is reduced compared to before? | Yes | No |
| Intermittent stream – do you stop and start more than once when you urinate? | Yes | No |
| Straining to void – do you have to push or strain to pass urine? | Yes | No |
| Terminal dribbling – at the end of your urination, do you dribble? | Yes | No |
| Postmicturition dribbling – do you dribble urine straight after you’ve finished urinating? | Yes | No |
| Double voiding – do you sometimes need to go twice in a short timeframe eg 5min apart | Yes | No |
| Incomplete emptying – do you feel like you have **not** emptied your bladder fully? | Yes | No |
|  | | |
| **Pain symptoms** | **Some** | **None** |
| Bladder pain on filling – do you experience any bladder pain/discomfort when it is full? | Yes | No |
| Bladder pain relieved by voiding – is this pain/discomfort relieved after emptying? | Yes | No |
| Bladder pain partially relieved by voiding – is this relieved slightly after emptying? | Yes | No |
| Bladder pain unrelieved by voids – is this not relieved after emptying? | Yes | No |
| Bladder or suprapubic pain – do you suffer from pain in the bladder area? | Yes | No |
| Loin pain – do you suffer from pain in the kidney area? | Yes | No |
| Dysuria – do you suffer from pain during urination in urethral area? | Yes | No |
| Urethral pain – do you suffer from pain in the urethral area? | Yes | No |
| Pain or discomfort referred to genitals? – do you have pain going to the genital area? | Yes | No |
| Left or right iliac fossa pain – do you have pain in the lower part of your tummy? | Yes | No |
| Pain radiating to legs – do you have pain going down the tops of your thighs? | Yes | No |
| Bladder pain during micturition – do you have pain while passing urine? | Yes | No |
| Pain after micturition – do you have pain after urinating? | Yes | No |
